# High temperature stress during flowering and grain filling offsets beneficial impact of elevated CO_2_ on assimilate partitioning and sink-strength in rice

**DOI:** 10.1038/s41598-017-07464-6

**Published:** 2017-08-15

**Authors:** Ashish K. Chaturvedi, Rajeev N. Bahuguna, Divya Shah, Madan Pal, S. V. Krishna Jagadish

**Affiliations:** 1 Division of Plant Physiology, Indian Agricultural Research Institute, New Delhi, 110012 India; 2 International Rice Research Institute, DAPO Box. 7777, Metro Manila, Philippines; 3 Department of Agronomy, Kansas State University, Throckmorton Center, Manhattan, Kansas 66506 United States of America

## Abstract

Elevated [CO_2_] (e[CO_2_]) environments have been predicted to improve rice yields under future climate. However, a concomitant rise in temperature could negate e[CO_2_] impact on plants, presenting a serious challenge for crop improvement. High temperature (HT) stress tolerant NL-44 and high yielding basmati Pusa 1121 rice cultivars, were exposed to e[CO_2_] (from panicle initiation to maturity) and a combination of e[CO_2_] + HT (from heading to maturity) using field based open top chambers. Elevated [CO_2_] significantly increased photosynthesis, seed-set, panicle weight and grain weight across both cultivars, more prominently with Pusa 1121. Conversely, e[CO_2_] + HT during flowering and early grain filling significantly reduced seed-set and 1000 grain weight, respectively. Averaged across both the cultivars, grain yield was reduced by 18 to 29%. Despite highly positive response with e[CO_2_], Pusa 1121 exposure to e[CO_2_] + HT led to significant reduction in seed-set and sink starch metabolism enzymatic activity. Interestingly, NL-44 maintained higher seed-set and resilience with starch metabolism enzymes under e[CO_2_] + HT exposure. Developing rice cultivars with higher [CO_2_] responsiveness incorporated with increased tolerance to high temperatures during flowering and grain filling using donors such as NL-44, will minimize the negative impact of heat stress and increase global food productivity, benefiting from [CO_2_] rich environments.

## Introduction

Carbon dioxide and temperature are major drivers of climate change affecting global food production. In spite of the challenges faced due to changing climate, global food production needs to increase by about 70% by 2050, to feed the growing population^1^. Rice is the most important cereal feeding more than 3 billion people globally and contributes about 20% of the total calorie intake by humans. Rice is majorly produced and consumed in Asia where it accounts for up to 80% of the caloric requirement^2^. Elevated [CO_2_] (e[CO_2_])could enhance rice yield through increased photosynthesis^3, 4^, improved water use efficiency^5^ and lower photo-respiratory losses^6^. However, competing energy requirement of carbon and nitrogen assimilation in plants leads to higher tissue C: N ratio under e[CO_2_], decreasing grain protein and other associated quality parameters in rice^7, 8^.

Higher rice yield under long term e[CO_2_] has been defined as a cumulative product of increased tillers, panicles, spikelets panicle^−1^, proportion of filled spikelets and 1000 grain weight^3, 9, 10^. Moreover, e[CO_2_] led to photosynthetic carbon gain and enhanced carbohydrate metabolism enzymatic activity in the source (leaf) tissue under non-stress conditions resulting in higher non-structural carbohydrate (NSC) accumulation in sink (seeds) among crop plants^11–15^. Interestingly, photosynthesis and net primary production under e[CO_2_] is directly related to sink efficiency in utilizing or storing the additional NSC, which could otherwise lead to photosynthetic acclimation in the source tissue^16^. Hence, cultivars with enhanced sink size and strength to store or utilize photo-assimilate could benefit under e[CO_2_] by minimizing photosynthetic acclimation and maintaining higher leaf level productivity^10, 17, 18^. Conversely, heat stress exposure during reproductive and grain filling stage is documented to reduce rice yield by reducing the proportion of fertile spikelets^19–21^, shorter grain filling period^22^ and loss of sink activity^23^. Additionally, leaf senescence, leading to net photosynthesis reduction^23, 24^ and lower sucrose-starch conversion enzymatic activity under heat stress is known to reduce final grain weight in rice^22^ and other crops including wheat^25^ and maize^26^.

The predicted increase in global temperature could be catastrophic to rice yield and quality when temperatures beyond known critical threshold (33 °C) coincide with sensitive growth and developmental stages^20^. Despite season long positive impact of e[CO_2_], post-heading heat stress could offset the e[CO_2_] effect on rice yield components and quality traits^27, 28^. Heat stress during grain filling leads to reduced utilization of additional NSC in the sink despite increased assimilate supply from leaves, under e[CO_2_]. For example heat stress resulted in reduced grain weight or grain starch content even with higher sucrose supply from source tissue under e[CO_2_] in rice^29, 30^. Recent studies reveal that rice plants grown under e[CO_2_] were more sensitive to heat stress compared to plants grown under ambient conditions^31^. Alternatively e[CO_2_] failed to compensate for the negative impact of heat stress on yield^32^ suggesting temperature to be the major driver inducing damage under e[CO_2_] + HT interaction. Negative impact of temperature documented on sink size and sink activity/strength (ability to utilize enhanced assimilate supply) in crop plants has instigated the need to explore crop responses to e[CO_2_] + HT interaction under realistic environmental conditions. Previous studies quantifying e[CO_2_] + HT interaction in rice are mostly conducted under controlled chamber conditions^15, 27, 29–31^ which would not translate into delineating crop responses under realistic field conditions, due to limitation in space, light etc^33^.

To date, dissecting the physiological and biochemical basis of sink regulation in rice under e[CO_2_] + HT stress interaction in tropical field conditions has not been attempted. A systematic analysis of e[CO_2_] + HT stress interaction was carried out at flowering and grain filling stage using two rice cultivars genetically differing in the spikelets number per panicle (sink size). A popular high yielding basmati rice cultivar Pusa 1121 with relatively smaller sink^34^ and a high yielding temperature tolerant rice cultivar NericaL-44 (NL-44) with larger sink size were studied with major emphasis on quantifying reproductive success and sink regulation, under e[CO_2_] + HT stress interaction under field conditions. Major objectives of the studies were to determine (i) photosynthetic attributes and assimilate partitioning to developing sink (grain) (ii) impact on key enzymes that determine sink-strength and yield attributes under e[CO_2_] + HT stress interaction using field based open top chambers.

## Results

### Growth environment

Meteorological observations in different experiments across both years are presented in Table 1 and Supplementary Fig. S1. In 2013, daily average a[CO_2_] concentration was 384.2 ppm (SD ± 11.5) while average e[CO_2_] treatment recorded 582.1 ppm (SD ± 49.9) and 569.3 ppm (SD ± 48.2), 571.5 ppm (SD ± 46.5) in 2013 and 2014, respectively. The day time and night time temperature and relative humidity (RH) across the replicate OTCs with a[CO_2_] and e[CO_2_] treatments, did not vary significantly, with an exception of temperature and RH being slightly higher with e[CO_2_] in 2013. During 2014, average day time temperature 33.6 °C (SD ± 3.3) and relative humidity 62% (SD ± 10.7) were comparable across all the OTCs with e[CO_2_] except an average higher temperatures of 3.5 °C (SD ± 1.2) above ambient recorded for e[CO_2_] + high temperature stress (e[CO_2_] + HT) treatment from heading until physiological maturity (Table 1; Supplementary Fig. S1).

**Table 1:** Carbon dioxide concentration, temperature and relative humidity (mean ± SD) inside OTCs during 2013 and 2014. Elevated [CO_2_] was imposed from panicle initiation until maturity (during the day time), while heat treatment was imposed from heading until maturity. ∆ indicates difference in respective parameter within the experiment. a[CO_2_] – ambient **[**CO_2_], e[CO_2_] – elevated **[**CO_2_] and e[CO_2_] + HT– elevated **[**CO_2_] + heat treatment. The data shown for 2014 is from heading to physiological maturity, before this period there were no significant difference in the environmental parameters.

Environmental Parameters	2013			2014		
	a[CO]_2_	e[CO_2_]	∆	e[CO_2_]	e[CO_2_] + HT	∆
[CO_2_] concentration (ppm)	384.2 ± 11.5	582.1 ± 49.9	197.9	569.3 ± 48.2	571.5 ± 46.5	2.2
Day temperature (°C) (0700 to 1900 h)	30.9 ± 4.9	31.9 ± 5.1	1.1	33.6 ± 3.3	37.1 ± 3.0	3.5
Night temperature (°C) (2000 to 0600 h)	25.6 ± 4.5	25.8 ± 4.4	0.2	27.8 ± 3.4	28.7 ± 3.5	0.9
Day relative humidity (%) (0700 to 1900 h)	77.1 ± 12.5	80.0 ± 11.8	2.9	61.9 ± 10.7	65.0 ± 11.1	3.1
Night relative humidity (%) (2000 to 0600 h)	86.1 ± 9.3	86.5 ± 9.3	0.4	79.1 ± 6.6	80.5 ± 7.1	1.4

### Yield and Yield parameters

Neither tiller numbers nor number of panicles hill^−1^ differed significantly with treatments or treatment x cultivar interaction in both years (Table 2). Elevated [CO_2_] significantly (P < 0.05) increased panicle weight hill^−1^ (12–18%) and grain yield hill^−1^ (15–24%) across cultivars during 2013. Although, Pusa1121 had higher gain under e[CO_2_], NL-44 showed higher panicle weight hill^−1^ and grain weight hill^−1^ across the treatments. Conversely, e[CO_2_] + HT significantly (P < 0.001) reduced panicle weight hill^−1^ (11–26%) and grain weight hill^−1^ (18–29%) across the cultivars compared to e[CO_2_] alone with Pusa 1121 recording higher reductions for both traits (Table 2). Significant cultivar (P < 0.001) and treatment (P < 0.01) effect was recorded for seed-set in 2013. NL-44 recorded significantly higher (increased by 10%) seed-set compared to Pusa 1121 (increased by 5%) under e[CO_2_] over a[CO_2_]. Conversely, a significant cultivar x treatment interaction (P < 0.05) was noted for seed-set in 2014. Seed-set reduced significantly under e[CO_2_] + HT (8 to 17%) averaged across cultivars compared to e[CO_2_]. However, reduction in seed-set under e[CO_2_] + HT was more prominent in Pusa 1121 (17%) (Table 2). A significant cultivar (P < 0.001) and treatment (P < 0.001) effect was noted for 1000 grain weight in 2013, with e[CO_2_] increasing 1000 grain weight by 3.6 and 6.6% in NL-44 and Pusa 1121, respectively compared to a[CO_2_]. A highly significant cultivar x treatment effect (P < 0.001) was noted for 1000 grain weight in 2014, with Pusa 1121 recording 17% and NL-44 recording only 3.2% lower 1000 grain weight under e[CO_2_] + HT compared to e[CO_2_](Table 2). Significant cultivars (P < 0.01) and treatment (P < 0.001) effect was noted for total biomass. NL-44 recorded higher biomass compared to Pusa 1121 across the treatments in 2013 with 13–15% increase in total biomass under e[CO_2_] averaged across the cultivars (Table 2). Total biomass decreased significantly under e[CO_2_] + HT compared to e[CO_2_] alone with a higher decline recorded in Pusa 1121 (24%) compared to NL-44 (11%) (Table 2).

**Table 2:** Grain yield and yield parameters in rice cultivars NL-44 and Pusa 1121 exposed to ambient and elevated [CO_2_] during 2013 and elevated [CO_2_] and elevated [CO_2_] + HT interaction during 2014. All the parameters were obtained at physiological maturity.

Year	Cultivars (C)	Treatments (T)	Number of tillers hill^−1^	Number of panicles hill^−1^	Panicle weight (g hill^−1^)	Grain yield^†^ (g hill^−1^)	Total number of spikelets panicle^−1^	Seed-set (%)	1000 Grain weight (g)	Total biomass (g hill^−1^)
2013	NL-44	a[CO_2_]	8.7 ± 0.4	8.3 ± 0.4	38.5 ± 1.6	29.9 ± 2.4	233.6 ± 6.8	82.8 ± 1.5	23.2 ± 0.2	66.4 ± 2.1
		e[CO_2_]	9.4 ± 0.5	9.2 ± 0.5	43.1 ± 2.8	34.5 ± 2.5	236.4 ± 6.1	87.3 ± 1.6	24.1 ± 0.5	75.2 ± 3.5
	Pusa 1121	a[CO_2_]	12.9 ± 0.8	12.1 ± 0.7	26.3 ± 1.4	20.3 ± 1.4	126.8 ± 2.5	71.7 ± 2.6	24.2 ± 0.0	58.8 ± 3.4
		e[CO_2_]	13.4 ± 0.6	12.3 ± 0.6	31.0 ± 1.5	25.1 ± 1.7	124.2 ± 5.7	79.1 ± 1.2	25.8 ± 0.1	67.3 ± 2.8
	LSD at 5%	C	1.2***	1.1***	3.8***	4.1***	11.7***	3.9***	0.6***	6.0**
		T	ns	ns	3.8*	4.1*	ns	3.9**	0.6***	6.0***
		C x T	ns	ns	ns	ns	ns	ns	ns	ns
2014	NL-44	e[CO_2_]	10.1 ± 0.5	9.5 ± 0.4	41.1 ± 1.4	35.9 ± 2.0	239.6 ± 2.1	83.5 ± 0.8	23.9 ± 0.2	69.8 ± 2.4
		e[CO_2_] + HT	9.3 ± 0.6	8.9 ± 0.5	36.5 ± 1.3	29.5 ± 1.4	235.0 ± 3.3	77.1 ± 0.9	23.1 ± 0.2	62.0 ± 1.6
	Pusa 1121	e[CO_2_]	13.5 ± 0.9	12.8 ± 0.8	30.4 ± 0.8	22.2 ± 0.5	124.0 ± 1.7	74.4 ± 1.4	25.7 ± 0.2	65.6 ± 2.3
		e[CO_2_] + HT	14.4 ± 0.5	13.5 ± 0.5	22.5 ± 0.6	15.9 ± 0.3	120.2 ± 3.4	61.7 ± 1.5	21.4 ± 0.3	48.8 ± 1.3
	LSD at 5%	C	1.3***	1.1***	2.2***	2.5***	5.8***	2.6***	ns	3.9***
		T	ns	ns	2.2***	2.5***	ns	2.6***	0.5***	3.9***
		C x T	ns	ns	ns	ns	ns	3.6*	0.7***	5.6*

Significance level: *P < *0.05*, **P < *0.01*, ***P < *0.001*, ns = non significant [LSD = least significant difference; HT = heat stress; a[CO_2_] = ambient CO_2_; e[CO_2_] = elevated CO_2_].

^**†**^Chaturvedi *et al*. 2017-Published grain yield data is used for associating the response mechanisms to the agronomic performance.

### Gas exchange parameters under e[CO_2_] and e[CO_2_] + HT interaction

**Figure 1:**
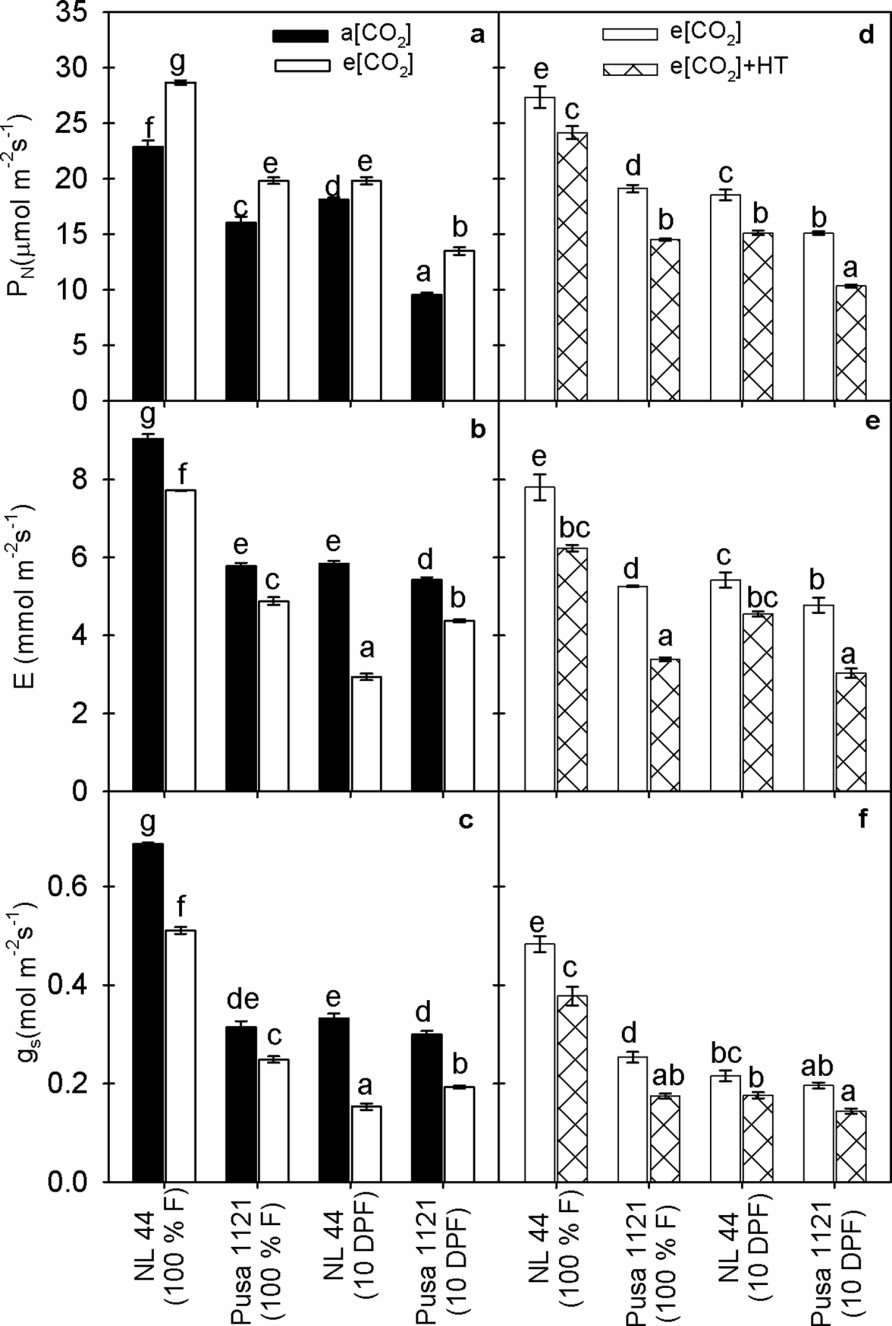
Changes in gas exchange parameters in flag leaf of rice cultivars NL-44 and Pusa-1121 at 100% flowering (100% F) and at 10 days post 100% flowering (10 DPF) under ambient and elevated [CO_2_] during 2013 (**a**–**c**) and elevated [CO_2_] and elevated [CO_2_] + HT environment during 2014 (**d**–**f**). Each vertical column represents mean of five replicates. Bars indicate ± SE. Comparison of means was obtained from Tukey’s honest significant difference test individually for 2013 and 2014 experiments. Means with the same letter are not significantly different at 5%. [P_N_ = photosynthetic rate; E = transpiration rate; g_s_ = stomatal conductance; HT = high temperature stress].

A significant cultivar x treatment x growth stage interaction (P < 0.001) was recorded for photosynthesis (P_N_) during 2013 (Supplementary Table S1). NL-44 recorded significantly higher P_N_ across cultivars, treatments and growth stages. Under e[CO_2_], both the rice cultivars recorded 24–25% gain in P_N_ at 100% flowering while Pusa 1121 recorded higher increase in P_N_ (41%) compared to NL-44 (9%) at 10 DPF (Fig. 1a). A significant cultivar, treatment and growth stage (P < 0.001) effect was recorded for transpiration rate (E) and stomatal conductance (g_s_) (Supplementary Table S1). NL-44 recorded highest E and g_s_ under a[CO_2_] at 100% F (Fig. 1b,c). Both E and g_s_ decreased significantly (P < 0.001) under e[CO_2_] across the cultivars (Fig. 1b,c). Moreover, NL-44 recorded a higher reduction (15 to 50%) in E compared to Pusa 1121 (16 to 20%). Similarly, e[CO_2_] reduced g_s_ by 26 to 54% in NL-44 and 21 to 36% in Pusa 1121 across the growth stages. Elevated [CO_2_] mediated reduction in E and g_s_ was higher at 10 DPF across the cultivars (Fig. 1b,c). During 2014, a significant cultivar, treatment and growth stage (P < 0.001) effect was noted for P_N_, E and g_s_ (Supplementary Table S1). Elevated [CO_2_] + HT caused significant (P < 0.001) decline in P_N_ (12 to 32%) compared to e[CO_2_] alone. Under e[CO_2_] + HT, Pusa 1121 and NL-44 recorded 24–32% and 12–19% reduction in P_N_ across the growth stages, respectively, compared to e[CO_2_] alone (Fig. 1d). Moreover, e[CO_2_] + HT significantly (P < 0.001) reduced E and g_s_ across the cultivars and growth stages (Supplementary Table S1). On average, the decline in both E and g_s_ was prominent in Pusa 1121 at 100% F (36% and 31%) and 10 DPF (37% and 27%), respectively compared to e[CO_2_] alone (Fig. 1e,f). Elevated [CO_2_] significantly (P < 0.001) increased leaf intrinsic water use efficiency (P_N_/g_s_) as compared to ambient [CO_2_] across the cultivars with highest increase recorded in NL-44 at 10 DPF (Supplementary Fig. S2a). Conversely, e[CO_2_] + HT treatment did not affect P_N_/g_s_ ratio significantly across the cultivars and growth stages (Supplementary Fig. S2b).

### Total soluble sugars (TSS) and starch content under e[CO_2_] and e[CO_2_] + HT interaction

**Figure 2:**
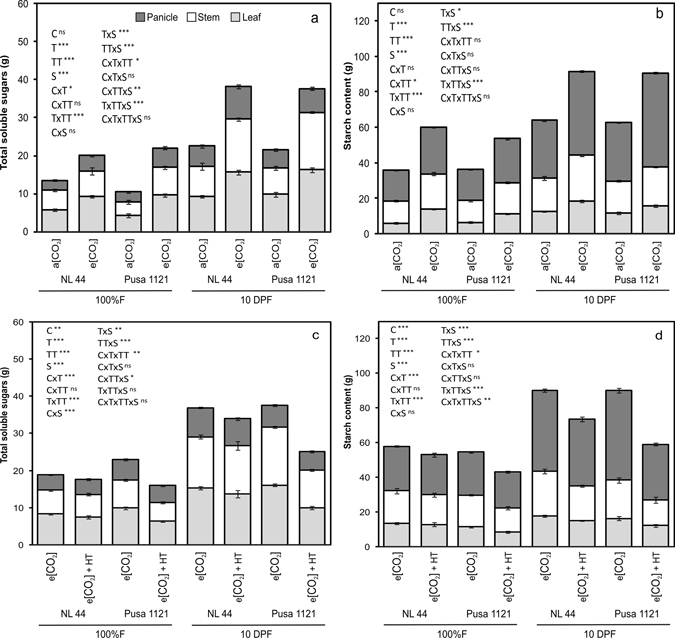
Changes in total soluble sugars and starch content in different plant parts (leaf, stem and panicle) of rice cultivars NL-44 and Pusa 1121 at 100% flowering (100% F) and at 10 days post 100% flowering (10 DPF) under ambient and elevated [CO_2_] during 2013 (**a**,**b**) and elevated [CO_2_] and elevated [CO_2_] + HT environment during 2014 (**c**,**d**). Each box in vertical stacked column represents mean of three replicates. Bars indicate ± SE. Asterisks indicates level of significance *P < *0.05*, **P < *0.01*, ***P < *0.001*, ns = non significant. [C = cultivar; T = treatment; TT = tissue type; S = growth stage].

A significant treatment x tissue type x growth stage (P < 0.001) effect was observed for TSS and starch content during 2013. However, cultivar response was non-significant for both the traits (Supplementary Table S2). At 10 DPF, higher TSS recorded in panicle (60%, 33%), stem (78%, 115%) and leaves (69%, 66%) for NL-44 and Pusa 1121 under e[CO_2_], respectively, a phenomenon that was similar at 100% flowering (Fig. 2a). Similarly, starch content increased significantly (P < 0.001) under e[CO_2_] across the cultivars, tissue type and growth stages (Fig. 2b). At 10 DPF, higher starch accumulation was recorded in panicle (45%, 60%), stem (38%, 21%) and leaves (45%, 35%) for NL-44 and Pusa 1121 under e[CO_2_] compared to a[CO_2_] (Fig. 2b).

A significant cultivar x treatment x tissue type interaction (P < 0.01) was noted for TSS while a significant cultivar x treatment x tissue type x stage interaction (P < 0.01) effect was recorded for starch content in 2014 (Supplementary Table S2). Elevated [CO_2_] + HT effect was more prominent at 10 DPF reducing TSS and starch content across the cultivars and tissue type (Fig. 2c,d). Pusa 1121 recorded a higher decline in stem (35%) and leaf (38%) TSS under e[CO_2_] + HT compared to e[CO_2_] alone at 10 DPF (Fig. 2c). Similarly, e[CO_2_] + HT significantly reduced panicle and stem starch content across the cultivars but with a higher reduction in Pusa 1121. Under e[CO_2_] + HT, panicle starch content was reduced by 17% and 38% for NL-44 and Pusa 1121, respectively, as compared to e[CO_2_] alone at 10 DPF (Fig. 2d).

### Sink strength regulating enzymes under e[CO_2_] and e[CO_2_] + HT interaction

**Figure 3:**
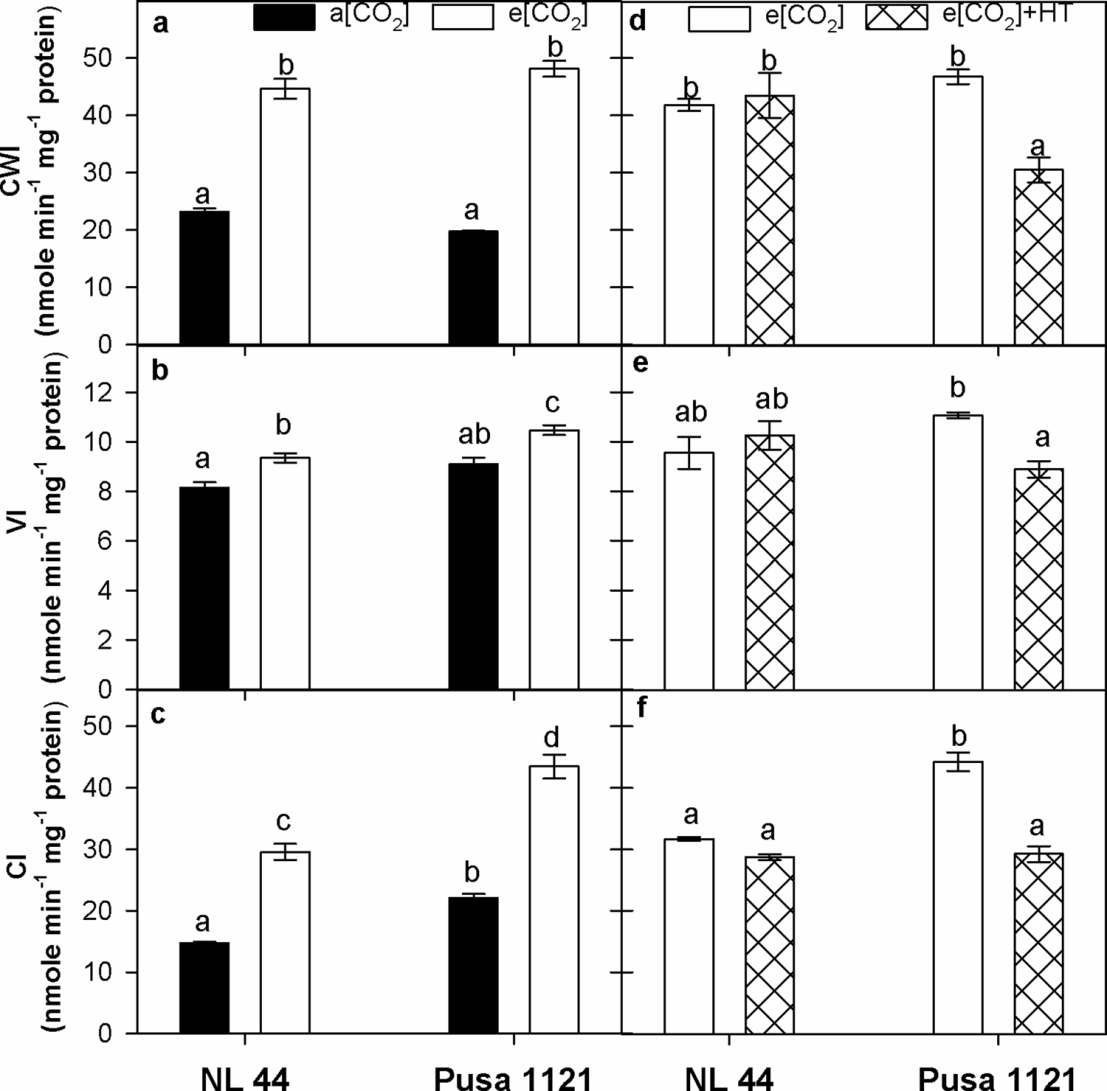
Cell wall invertase (**a**,**d**), vacuolar invertase (**b**,**e**) and cytosolic invertase (**c**,**f**) activity in spikelets of rice cultivars NL-44 and Pusa 1121 at 10 days post 100% flowering (10 DPF) under ambient and elevated [CO_2_] during 2013 (**a**–**c**) and elevated [CO_2_] and elevated [CO_2_] + HT environment during 2014 (**d–f**). Each vertical column represents mean of three replicates. Bars indicate ± SE. Comparison of means was obtained from Tukey’s honest significant difference test individually for 2013 and 2014 experiments. Means with the same letter are not significantly different at 5%. [CWI = cell wall invertase; VI = vacuolar invertase; CI = cytosolic invertase].

During 2013, a significance treatment (P < 0.001) effect was noted for CWI, VI and CI activity, with a cultivar x treatment interaction (P < 0.05) for CWI and CI activity (Supplementary Table S3). Elevated [CO_2_] significantly (P < 0.001) increased CWI activity (93 to 144%), VI activity (15%) and CI activity (97 to 101%) across the cultivars as compared to a[CO_2_](Fig. 3a–c). During 2014, a significant cultivar x treatment effect (P < 0.05 to 0.001) was noted for CWI, VI and CI activity (Supplementary Table S3). Invertase(s) enzymes activity did not significantly change in NL-44 under e[CO_2_] + HT, while Pusa 1121 recorded a significant decline (20 to 35%) in all three invertases activity under e[CO_2_] + HT compared to e[CO_2_]treatment (Fig. 3d–f).

**Figure 4:**
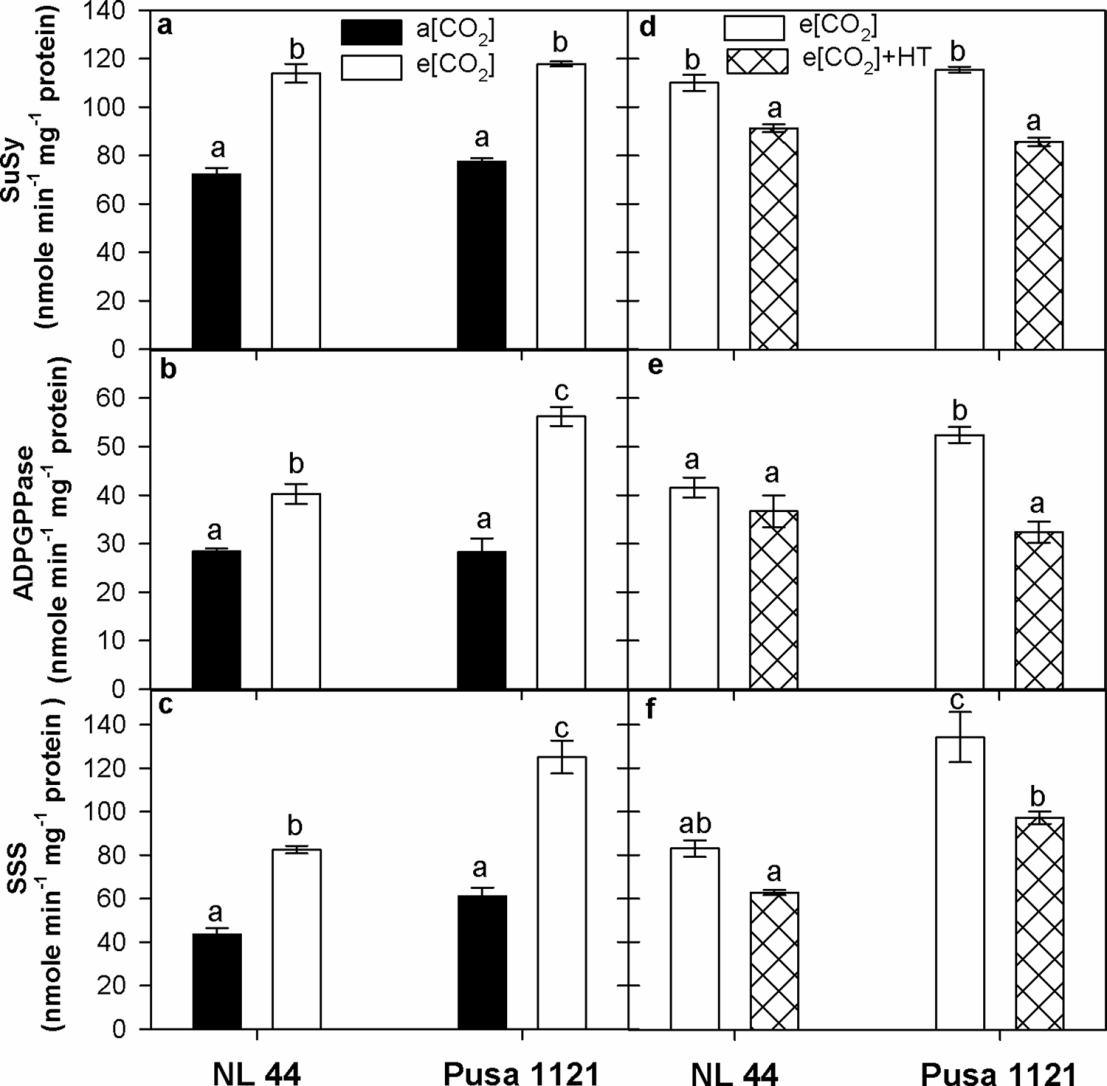
Sucrose synthase (**a**,**d**), ADP glucose pyrophosphorylase (**b**,**e**) and soluble starch synthase (**c**,**f**) activity in spikelets of rice cultivars NL-44 and Pusa 1121 at 10 days post 100% flowering (10 DPF) under ambient and elevated [CO_2_] during 2013 (**a**–**c**) and elevated [CO_2_] and elevated [CO_2_] + HT environment during 2014 (**d**–**f**). Each vertical column represents mean of three replicates. Bars indicate ± SE. Comparison of means was obtained from Tukey’s honest significant difference test individually for 2013 and 2014 experiments. Means with the same letter are not significantly different at 5%. [SuSy = sucrose synthase; ADPGPPase = ADP glucose pyrophosphorylase; SSS = soluble starch synthase].

A significant treatment effect (P < 0.001) was recorded for SuSy activity (Supplementary Table S3). Elevated [CO_2_] increased SuSy activity (52 to 58%) across the cultivars compared to a[CO_2_] (Fig. 4a). A significant cultivar x treatment effect (P < 0.05 to 0.01) was observed for ADPGPPase and SSS activity (Supplementary Table S3). Both the enzymes recorded 42 to 104% increase under e[CO_2_] across the cultivars. However, Pusa 1121 recorded higher ADPGPPase and SSS activity than NL-44 under e[CO_2_] compared to a[CO_2_] (Fig. 4b,c). During 2014, a significant cultivar x treatment effect (P < 0.05 to 0.01) was observed for SuSy and ADPGPPase activity while significant cultivar (P < 0.001) and treatment (P < 0.01) effect was recorded for SSS activity (Supplementary Table S3). SuSy activity reduced significantly (P < 0.05) in NL-44 (17%) and Pusa 1121 (26%) under e[CO_2_] + HT treatment compared to e[CO_2_] (Fig. 4d). Conversely, ADPGPPase and SSS activity under e[CO_2_] + HT treatment was reduced by 38 and 28% in Pusa 1121 compared to e[CO_2_] treatment. NL-44 did not show significant change in ADPGPPase and SSS activity across the treatments (Fig. 4e,f).

## Discussion

The green revolution dramatically increased cereal crop yields including rice, driven by modern agricultural inputs such as improved varieties and fertilizers^35^. Despite several technological interventions and breeding efforts after the green revolution, global rice production is either reaching a plateau or in some cases dropping^36^. Elevated [CO_2_] has been documented to increase productivity in C3 plants including rice by providing more carbon to be fixed to biomass and yield^5, 17, 37^. However, a concomitant increase in temperature could negate this beneficial effect particularly if high temperature coincides with critical developmental stages such as flowering^27^. Increase in spikelet density, biomass with higher tiller and panicle number are major traits influenced by e[CO_2_] at vegetative and early reproductive stage in rice^3, 9^. However, vegetative stage is relatively tolerant to high temperature^38^ and heat stress effects are more confined to impacting flowering negatively, reducing reproductive success and seed-set^20, 21, 38^, and at ripening stage affecting grain filling rate and duration^22, 39^. Thus, it is important to quantify the effect of e[CO_2_] on the traits determining yield at temperature sensitive reproductive and grain filling stage in rice, without confounded by the impact of e[CO_2_] on tiller, panicle and spikelet numbers. Considering higher sensitivity of reproductive and grain filling stage to heat stress, we have for the first time systematically analyzed the mechanistic response of e[CO_2_] effect on key traits regulating yield at flowering and early grain filling stage. Additionally, the counter impact of high temperature stress on these traits and mechanisms negating positive advantage of e[CO_2_] in rice is examined under realistic field conditions using OTC facility.

Elevated [CO_2_] exposure from reproductive (starting at agronomic panicle initiation stage) until grain filling and maturity did not change tiller number hill^−1^, panicle number hill^−1^ and spikelets panicle^−1^ (Table 2). It has been documented that tiller number, panicle number and spikelets panicle^−1^ are mostly determined during early panicle initiation stage^40^, supporting previous findings. Thus, e[CO_2_] exposure starting subsequent to panicle initiation stage had minimum influence on these traits. Conversely, panicle weight hill^−1^, grain yield hill^−1^, 1000 grain weight and total biomass hill^−1^ were significantly increased under e[CO_2_] (Table 2). Yield enhancement in crops by 10–20% has been documented under 550–600 µmolmol^−1^compared to 380–400 µmol mol^−1^ CO_2_^41, 42^. We observed increase in current photosynthetic rate under e[CO_2_] (Fig. 1a) and thus more carbon fixed to carbohydrates (Fig. 2). Besides higher photosynthesis, lower transpiration rate and stomatal conductance recorded under e[CO_2_] (Fig. 1b,c), resulted in higher intrinsic water use efficiency (P_N_/g_s_) across the cultivars (Supplementary Fig. S2). Our findings supports earlier findings with e[CO_2_]^4, 5, 10, 17^. Higher accumulation of total soluble sugars and starch under e[CO_2_] were documented from stem and panicles across both the cultivars (Fig. 2a,b). Stem is a major sink for photo-assimilates during vegetative and early reproductive phase^43^. However, developing grains become major sink immediately after the onset of grain filling, for both stored assimilates in the stem and current photo-assimilates being produced majorly by top three leaves in rice^44–46^. Yoshida *et al*.^44^ reported that accumulated stem carbohydrates until heading can contribute up to 40% while photosynthesis during grain filling contributes up to 60% of grain carbohydrate under most conditions. Significantly higher sucrose content recorded in the stem and starch content in the panicle (Fig. 2a,b) helped to increase panicle weight and grain weight across both the cultivars (Table 2). Accumulation of starch in the developing grains is majorly determined by efficient translocation of photo-assimilate to sink organ and its ability to utilize the transported assimilate (sink strength) for producing storage sugars (starch) in the endosperm^47^. Cell wall invertase and sucrose synthase play major role in determining sink strength in the developing caryopsis^47–49^. Cell wall invertase unloads transported sucrose into apoplast of sink tissue by breaking down sucrose in to glucose and fructose, which eventually lead to formation of ADP/UDP glucose and fructose by sucrose synthase, which is the first step for starch production through starch synthase in the endosperm^50^. Interestingly, we observed a significant influence of e[CO_2_] on sink strength regulating enzymes (Figs 3 and 4). Elevated [CO_2_] increased CWI and SuSy activity across the cultivars clearly demonstrating e[CO_2_] influence on sink strength facilitating more assimilate transport and conversion to starch in the developing grain, supporting higher starch accumulation in the panicles at 10 DPF (Fig. 2b). Beside CWI, VI plays a major role in sink initiation and expansion by supporting cell division during pre-storage phase in the grain while limited information is available on the physiological function of CI^47, 49, 51^. Elevated [CO_2_] increased ADPGPPase and SSS activity, which are key enzymes for starch synthesis (Fig. 4). ADPGPPase plays a crucial role in starch synthesis by producing ADP-glucose used by starch synthase for starch synthesis. Thus, both SuSy and ADPGPPase could provide substrate (UDP/ADP-glucose) for starch synthesis in the endosperm^52, 53^. Moreover, ADPGPPase helps in recycling grain starch by synthesizing ADP-glucose from glucose units derived from starch breakdown in the endosperm^53^. Conversely, SSS synthesize starch and a positive correlation of SSS activity with starch synthesis has been documented in wheat^54^ and rice^55, 56^ grains. Higher SSS activity under e[CO_2_] is in line with higher starch accumulation in the panicle (Fig. 2b). Thus, positive effect of e[CO_2_] on sink strength and starch synthesis enzymes could contribute in efficient translocation of photo-assimilate in to developing sink and conversion of sucrose to starch in the endosperm. Higher starch accumulation in the grains increased 1000 grain weight (Table 2), a major yield determining parameter as documented by studies involving crops including wheat and rice^57, 58^. Previous studies^10, 37, 59^ have reported an increase in filled spikelet numbers (seed set) under e[CO_2_] in rice, similar to our findings attributed to increased availability of sugars (sucrose) during reproductive development. Adequate import and efficient utilization of sucrose is required for gametophyte development, fertilization and coordinated development of maternal tissues, which collectively determines seed-set in crop plants^60^. Thus, the effect of e[CO_2_] during reproductive and grain filling stage influences an increase in total biomass, 1000 grain weight and proportion of filled spikelets collectively contributing to yield increase (Table 2).

As indicated earlier, future climatic scenarios with e[CO_2_] accompanied by higher temperature presents a unique challenge for crop improvement due to antagonistic interaction of these factors^31, 32^. Despite higher growth and yield, e[CO_2_] grown plants could be more sensitive to higher temperatures due to reduced stomatal conductance and transpiration under e[CO_2_]^31, 61^ (Fig. 1; Supplementary Fig. S2). This can potentially reduce plasticity to avoid high tissue temperature by limiting transpirational cooling, an effective strategy under high temperature^38, 62^. Sensitivity of rice reproductive stages to heat stress is well documented^19–21^ which can lead to significant yield loss due to lower spikelet fertility, seed-set and ultimately lowering harvest index^63^. Flowering and grain filling stages sensitivity to heat stress and resulting fertility and yield loss in rice is mostly documented from studies using controlled environment facilities^20–22, 39^_,_ with the presented results addressing  this knowledge gap on mechanisms leading to yield gain and loss with eCO_2_ and eCO_2_ + HT under realistic field conditions.

Interactive effect of e[CO_2_] + HT had a prominent and negative effect on seed-set, 1000 grain weight, total biomass hill^−1^, panicle weight hill^−1^ and grain weight hill^−1^ (Table 2). Number of filled grains is the most important yield component accounting for e[CO_2_] + HT effect^32, 64, 65^. We observed up to 17% decline in seed-set in Pusa1121 while NL-44 ability to maintain high fertility rate under severe heat stress under field conditions has been documented^38^. The greater negative impact on 1000 grain weight can be attributed to e[CO_2_] + HT exposure throughout the grain filling stage which significantly affected CO_2_ assimilation (Fig. 1d), assimilate partitioning (Fig. 2c,d) and activity of sink enzymes (Figs 3 d–f and 4d–f). The top three leaves including the flag leaf, contribute a significant portion of current photo-assimilate during grain filling stage^44–46^. Beside reducing current photo-assimilate supply during grain filling, lower stomatal conductance and transpiration (Fig. 1e,f) contributed to aggravate high temperature stress impact. We observed a significant decline in tissue total soluble sugars at flowering and panicle starch content at grain filling with a more prominent negative effect in Pusa 1121. This interesting observation highlighted potential of NL-44 as high temperature tolerant cultivar which was able to maintain higher fertility^38^ and panicle starch content under e[CO_2_] + HT, which is a novel finding. Reduced panicle starch content in Pusa 1121 is attributed to poor sink strength and starch synthesis under e[CO_2_] + HT (Figs 3 d–f and 4d–f). Despite a general increase in sink carbohydrate metabolism enzymes activity under e[CO_2_] across both the cultivars, contrasting response was observed under e[CO_2_] + HT treatment. The tolerant NL-44 was not significantly affected except SuSy, compared to Pusa1121 which recorded 25 to 35% decline across all the starch metabolism enzymes studied. Earlier reports suggest sensitivity of sink related enzymes under high day^22^ and high night temperature^47^ affecting starch accumulation and final grain weight. Additionally, reduced starch content in grains has been correlated with declined SSS activity under high temperature stress^66^. For the first time we report interactive effect of e[CO_2_] + HT on key sink strength enzymes activity which determine rice grain filling potential affecting grain weight and yield.

Our study concludes that e[CO_2_] has a significant and positive impact at reproductive and grain filling stage, translating to higher seed-set and improved sugar partitioning to the sink tissue (caryopsis) governed by sink enzymes. Higher photosynthetic rate during critical grain filling stage and starch accumulation in the panicle contributed to increased grain weight, a major contributing trait enhancing yield. Conversely, e[CO_2_] + HT treatment negated the positive impact of e[CO_2_] prominently in Pusa 1121 leading to significant decline in seed-set and lowered sink starch metabolism enzymes. Interestingly, NL-44 known for tolerance to heat stress helped in maintaining higher seed-set under e[CO_2_] + HT, validating previous findings and providing mechanistic evidence for its extended tolerance to high temperature stress even during grain filling. Developing rice cultivars with higher CO_2_ responsiveness incorporated with increased tolerance to high temperatures during flowering and grain filling using donors such as NL-44, will minimize the negative impact of heat stress and increase global food productivity, benefiting from CO_2_ rich environments.

## Materials and Methods

### Experimental setup

Field experiments were conducted during kharif season (July–October) in 2013 and 2014, using open top chambers (OTC) for temperature and e[CO_2_] exposure. All the experiments were carried out in field-based OTC facility at Indian Agricultural Research Institute (IARI), New Delhi, India (28^◦^35′N latitude, 77^◦^12′E longitude). Two cultivars Pusa 1121 (high yielding basmati) and Nerica L-44 (high yielding, high temperature tolerant^38^) were used. Both cultivars selected were of similar duration and phenology. Key growth stages, observations and sampling time points for both the cultivars are described in Supplementary Figure S1. During 2013, OTC facility was used to expose plants to e[CO_2_] from panicle initiation until maturity. Three OTCs (each 7.1 m^2^ area) were used for e[CO_2_] treatment and other three for ambient [CO_2_] (a[CO_2_]) to serve as control. Total area in each OTC was divided in two equal halves for transplanting Pusa 1121 and NL-44^28^. Twenty-one-day-old seedlings were transplanted on July 3^rd^ 2013. Nitrogen (urea), phosphorus (single superphosphate) and potassium (muriate of potash) were applied at the rate of 120, 40 and 60 kg ha^−1^, respectively. The entire dose of P and K fertilizers were applied as basal dose while N fertilizer was applied in three splits i.e., 50% a day before transplanting, 25% at active tillering and the remaining 25% at the booting stage^38^. All the plants were maintained under flooded soil with standing water 5 cm above soil surface from transplanting until a week before harvest.

### Elevated [CO_2_] exposure in OTCs

OTC facility used for [CO_2_] enrichment in the experiments is described in detail by Pal *et al*.^67^ and Saha *et al*.^68^. Briefly, the diameter and height of each OTC was 3.0 m and 2.5 m, respectively, with the upper end having a frustum of 0.5 m to maintain similar temperature and relative humidity within the OTC as ambient atmosphere. The OTC aluminum frame was lined with 125 μm thick polyvinyl chloride sheet with >85% transmittance of sunlight. Elevated [CO_2_] level (~200 ppm above ambient) was maintained by releasing pure [CO_2_] gas inside OTC from a commercial grade [CO_2_] cylinder. Each cylinder was fitted with a regulator (DURA, ESAB, India) through solenoid valves. The [CO_2_] concentration was monitored using a [CO_2_] sensor (NDIR, Topak, USA) fixed in the middle of each OTC at 1.5 m height from the ground. Real time basis [CO_2_] data logging, control and operation was performed by microprocessor through digital input and output module as described previously^67, 68^. Temperature and humidity were recorded at every 30 min interval inside all OTCs with the help of temperature and humidity sensors (Model TRH 511, Ambetronics, Switzerland). Each OTC surface was cleaned weekly to maintain maximum transparency throughout the experimental period.

### High temperature stress in combination with e[CO_2_]

In 2014, OTCs were used to quantify rice response to e[CO_2_] + HT interaction. Cultivars, crop management practices, [CO_2_] treatment and monitoring were identical to 2013. Out of six OTCs, three were used for e[CO_2_] exposure while remaining three OTCs were used for imposing e[CO_2_] + HT stress^28^. High temperature treatment was imposed during day time (0700 to 1900) using heat radiators (Oil filed radiator 3211 F PTC, Usha, India), starting from heading until maturity. Inbuilt temperature sensor within heat radiators, fitted with a thermostat automatically regulated and maintained temperature within the OTC at target levels of 3 to 4 °C above ambient. The air temperature and humidity inside each OTC was measured as described for 2013 experiment.

Elevated [CO_2_] effect on tiller number, panicle number and spikelet panicle^−1^ are determined during the pre-flowering stage. The beneficial effect of e[CO_2_] on these primary traits could confound analysis of e[CO_2_] either independently or in combination with heat stress during the heat sensitive reproductive and grain filling stage, which was major objective of the studies. Hence, we have confined e[CO_2_] treatment starting from panicle initiation till physiological maturity stage while e[CO_2_] + HT from heading to physiological maturity^28^.

### Sampling and data collection

#### Gas exchange measurements

Gas exchange measurements were recorded in flag leaf of five randomly selected plants at 100% flowering and mid grain filling (milky) stage (10 days post 100% flowering; DPF) across the treatments during 2013 and 2014. All the measurements were recorded by portable photosynthesis system LI-6400XT (LI-COR Inc., Lincoln, NE, USA) between 0900 to 1130 h. CO_2_ concentration of the sample chamber was controlled with the LI-COR CO_2_ injection system and a near-saturating photosynthetic photon flux density (PPFD) of 1200 µmol m^−2^ s^−1^, given from an inbuilt LI-6400XT LED light source. Air temperature of the sample chamber was maintained similar to OTC chamber temperature conditions. Relative humidity was controlled through desiccant and maintained between 65 to 80%. Prior to logging the data, the selected sample (leaf) were kept in the sample chamber until the instrument attained a steady state of photosynthetic carbon assimilation.

#### Yield and yield components

Plant samples were harvested at physiological maturity and from each replication, five hills were taken from the middle rows to avoid any confounding border effects. Number of tillers and panicles were counted manually from each hill. Panicles were separated from all the harvested samples and only straw was oven dried at 70 °C until constant weight was obtained. The panicles were sundried in net-bags and weighed using analytical balance (model: BSA124S-CW, Sartorius AG, Germany). Grain yield was determined for each hill and adjusted to the standard moisture content (0.14 g H_2_O g^−1^)^47^. The above-ground total biomass and 1000 grain weight was calculated using analytical balance. The above-ground total biomass was the combined dry matter of straw and sun dried panicles. Five random panicles were selected from each hill for calculating total number of spikelets per panicle and percent seed-set. Total number of spikelets was counted manually for each panicle. Seed-set was recorded after separating the filled and unfilled grains. The ratio of filled grains to total number of spikelets per panicle was estimated and expressed as a percentage^20^.

#### Non-structural Carbohydrates (NSC)

Plant samples from both the cultivars were harvested between 0800 and 1000 h at 100% flowering and mid grain filling stage (10 DPF) for estimating NSC (total soluble sugars and starch) content following Yoshida *et al*.^69^ and Shi *et al*.^70^. Five hills were harvested from each treatment in experiments in both the years. Plant samples were separated into leaves, stem and panicles and immediately treated with a heat burst in the microwave^71^ followed by oven drying at 70 °C for 48 h. The samples were then ground and 0.1 g of ground sample was taken for NSC estimation (% dry weight of tissue). In brief, ground leaf, stem and panicle samples were extracted using 80% ethanol (v/v). The extract was used for soluble sugar analysis after adding anthrone reagent. The residue remaining after soluble sugars extraction, was dried and further extracted using perchloric acid for starch analysis using the anthrone reagent. Absorbance of the samples was taken at 630 nm using UV-visible spectrophotometer (model: Specord Bio. 200, AnalytikJena, Germany).

#### Enzyme Extraction and Assay

Main tiller panicle from three randomly selected hills per cultivar and treatment were sampled in to liquid N and stored at −80 °C for further analysis. All samples for enzyme assays were collected at 10 DPF which is considered as suitable stage for studying starch synthesis and accumulation in rice grain^47, 72^.

#### Enzyme extraction

The enzyme extraction and assay for ADP Glucose Pyrophosphorylase (ADPGPPase^73^), invertases^74^; sucrose synthase (SuSy^75^) and soluble starch synthase (SSS^76^) were quantified from the spikelets detached and randomly selected from the frozen panicles. Specific activity of all the enzymes was expressed in per milligram protein. Protein concentration of the crude plant extract was estimated as per Bradford^77^.

Whole spikelets (0.4 g) were homogenized under liquid N_2_ in a pre-chilled mortar and pestle. The homogenate was collected in a 2.0-ml eppendorf tube and 1.5 ml of extraction buffer [50 mM Hepes-NaOH (pH 7.5), 1 mM Na_2_EDTA, 5 mM MgCl_2_, 0.5% bovine serum albumin (BSA) and 2.5 mM DTT for ADPG-PPase; 25 mM Hepes-NaOH (pH 7.5), 0.5 mM ethylene-diamine-tetra-acetic acid (EDTA), 5 mM MgCl_2_, 2% poly(ethylene glycol)-20, 1% BSA and 3 mM DTT for SuSy; 100 mM tricine-NaOH (pH 8.0), 2 mM EDTA,8 mM MgCl_2_, 12.5% (v/v) glycerol, 50 mM 2-mercaptoethanol, and 5%(w/v) insoluble polyvinylpyrrolidone-40 for SSS; 100 mM Hepes-KOH (pH 7.4), 1 mM EDTA, 5 mM MgCl_2_, 1 mM methylene glycol-bis(b-minoethylether)-N,N,N9,N9,-tetraacetic acid (EGTA), 1 mM phenylmethylsulphonyl-fluoride (PMSF), 5 mM diothiothreitol (DTT), 200 ml l^−1^ glycerol, 1 ml l^−1^ Triton X-100, and 5 mM thiourea for invertase] was added. The homogenate was gently mixed and centrifuged for 5 min at 14,000 g. Supernatant was directly used as the enzyme source for the SSS and ADPGPPase assay. Supernatant was desalted on a 3-ml Sephadex G-50 column (equilibrated with 25 mM Hepes-NaOH (pH 7.5), 0.5 mM EDTA and 5 mM MgCl_2_,) at 4 °C and desalted extract was used for the SuSy enzyme assay. For invertase, supernatant was retained for soluble invertase [cytosolic invertase (CI) and vacuolar invertase (VI)] assay. The pellet was washed once with extraction buffer (0.5 ml) and supernatant was retained after centrifuge as above. The washed pellet fraction was finally suspended in 1.8 ml extraction buffer for insoluble invertase (cell wall invertase, CWI) assay.

#### Enzyme assay

For ADPGPPase activity, reactions were assayed at 35–37 °C in a reaction mixture (500 µl) containing: 40 mM Hepes-NaOH (pH 7.5), 4 mM MgCl_2_, 0.1 mg BSA, 1 mM ADP glucose, 1 unit of phosphoglucomutase, 1 unit of glucose-6-P dehydrogenase, 0.3 mM NAD and 40 µl of enzyme extract. Sodium pyrophosphate (1 mM) was added when a plateau was reached at 340 nm (<5 min). The activity was calculated on the initial linear part of the time dependent increase in absorbance at 340 nm.

SuSy activity was estimated by formation of sucrose from UDP-glucose. The assay mixture (70 µl) containing 40 mM Hepes-NaOH (pH 7.5), 8 mM fructose, 8 mM UDP-glucose,15 mM MgC1_2_ and desalted extract. The assay mix was incubated for 10 min at 25 °C, and reactions were terminated by adding equal volume of 1 N NaOH. Unreacted fructose was destroyed by placing the tubes in a boiling-water bathfor 10 min. The tubes were cooled at room temperature and 0.25 ml of 1% resorcinol in ethanol and 0.75 ml of 30% HCl were added. The tubes were further incubated for 8 min at 80 °C and cooled on ice. Tubes were finally centrifuged for 5 min at 5000 g and absorbance was measured at 520 nm.

The SSS assay was performed in a 280-µl reaction mixture containing 50 mM Hepes-NaOH (pH 7.4), 0.7 mg amylopectin, 1.6 mM ADP glucose,15 mM DTT and enzyme extract. The reaction mixture was incubated for 20 min at 25 °C. Tubes with reaction mixture were put in a boiling-water bath for 30 s to inactivate the enzyme. Then, 100 µl of a solution of 50 mM Hepes-NaOH (pH 7.4), 4 mM phospho (enol) pyruvate, 10 mM MgCl_2_, 200 mM KC1 and pyruvate kinase (1.2 U) was added to reaction mixture and incubated further for 30 min at 30 °C. The mixture was heated in a boiling-water bath for 30 s and then subjected to centrifugation for 5 min at 10,000 g. The supernatant (300 µl) was mixed with a solution of 50 mM Hepes-NaOH (pH 7.4), 20 mM MgCl_2_,10 mM glucose and 2 mM NADP. The production of G6P from glucose was determined from the increase in absorbance at 340 nm after adding 1 µl each of G6P dehydrogenase (0.35 U) and hexokinase (1.4 U).

Extract (40 µl) was added on ice to a 180 µl assay mixture containing 0.1 M sucrose; and either 50 mM Bicine-KOH (*N,N-*bis[2-hydroxyethyl] glycine) pH 7.6 (for CI) or 50 mM sodium acetate at pH 4.7 and pH 4.3 for CWI and VI, respectively. At time zero, assays were transferred for 1 h to 30 °C in a water bath, and then for 3 min to 85 °C. Time zero controls were included, in which the incubation at 30 °C was omitted. Prior to heating at 85 °C, assays and controls for CWI and VI were alkalinized by the addition of 30 µl of 1 M Tris-HCl at pH 8.0. A 70 µl assay mixture was added to the 190-µl fructose assay mix (100 mM Hepes-KOH pH 7.4, 1.1 mM ATP,2.25 mM MgCl_2_, 0.2 U hexokinase and 1.1 mM NADP). The production of G6P from glucose was determined from the increase in absorbance at 340 nm and upon the addition of 0.2 unit (U) of NADP-dependent G6P dehydrogenase.

#### Statistical analysis

All data were analyzed using a two-way ANOVA comparing the effect of treatment and cultivar means (SPSS Inc., Chicago, USA). Tukey’s post-hoc test was used to separate treatment means. Unless otherwise stated, differences were determined as significant at the P ≤ 0.05 level.

## Electronic supplementary material

Supplementary Figures and Tables
