# Supplementary material for: High temperature stress during flowering and grain filling offsets beneficial impact of elevated CO2 on assimilate partitioning and sink-strength in rice
Source: Sci Rep. 2017 Aug 15;7:8227. doi: 10.1038/s41598-017-07464-6 (PMC5557921; doi:10.1038/s41598-017-07464-6)
Supplement: Supplementary file 1 — Supplementary Figures and Tables [file 41598_2017_7464_MOESM1_ESM.pdf]

Supplementary Information

Title: High temperature stress during flowering and grain filling offsets beneficial impact of elevated CO_2 on assimilate partitioning and sink-strength in rice

Author List Ashish K. Chaturvedi, Rajeev N. Bahuguna, Divya Shah, Madan Pal, Krishna S.V. Jagadish

This file includes

Supplementary Figure S1 and S2

Supplementary Table S1 to S3

Supplementary Figure S1. Temperature, relative humidity and rain fall data recorded for the rice experiments during 2013 and 2014. Elevated CO_2 exposure during both year experiments was initiated at panicle initiation and continued until physiological maturity. High temperature exposure during 2014 was initiated from heading until physiological maturity. Phenology of both the cultivars was similar except 1-2 days delay observed in heading of Pusa 1121 across the experiments and treatments. However, e[CO_2] and e[CO_2]+HT treatments resulted in earlier heading, flowering and physiological maturity across the cultivars or experiments. Dotted vertical line shows heading (H) date for both the cultivars as observed under a[CO_2] and e[CO_2], respectively. Vertical descending arrows denote 100% flowering (100% F) and 10 days post flowering (10 DPF) when gas exchange traits and biochemical sampling has been done across the cultivars and experiments. [Tavg.= average day time temperature (0700 to 1700 hrs); RH= average day time relative humidity; H= heading; 100% F= 100% panicle at flowering; 10 DPF= 10 days post 100% flowering].

Supplementary Figure S2. Changes in intrinsic water use efficiency (WUEi; P_N/g_s) in flag leaf of rice cultivars NL-44 and Pusa-1121 at 100% flowering (100 % F) and at 10 days post 100% flowering (10 DPF) under ambient and elevated [CO_2] during 2013 (a) and elevated [CO_2] and elevated [CO_2] + HT environment during 2014 (b). Each circle represents mean of five replicates. Bars indicate ± SE. Significance level: *$P<0.05$, ***$P<0.001$, ns=non significant. [C=cultivar; T= treatment; S= growth stage].

Supplementary Table S1. *ANOVA* table for leaf gas exchange parameters of rice cultivars NL-44 and Pusa 1121 measured at 100 % flowering (100% F) and 10 days post 100% flowering (10 DPF) under ambient and elevated [CO_2] during 2013 and elevated [CO_2] and elevated [CO_2] + HT environment during 2014. Values presented are least significant difference (LSD) for each trait.

Supplementary Table S2. *ANOVA* table for total soluble sugars and starch content from leaf, stem and panicle of rice cultivars NL-44 and Pusa 1121 measured at 100 % flowering (100% F) and 10 days post 100% flowering (10 DPF) under ambient and elevated [CO_2] during 2013 and elevated [CO_2] and elevated [CO_2] + HT environment during 2014. Values presented are least significant difference (LSD) for each trait.

Supplementary Table S3. *ANOVA* table for sink enzymes activity in the spikelets of rice cultivars NL-44 and Pusa 1121 measured at 100 % flowering (100% F) and 10 days post 100% flowering (10 DPF)

under ambient and elevated [CO_2] during 2013 and elevated [CO_2] and elevated [CO_2] + HT environment during 2014. Values presented are least significant difference (LSD) for each trait.

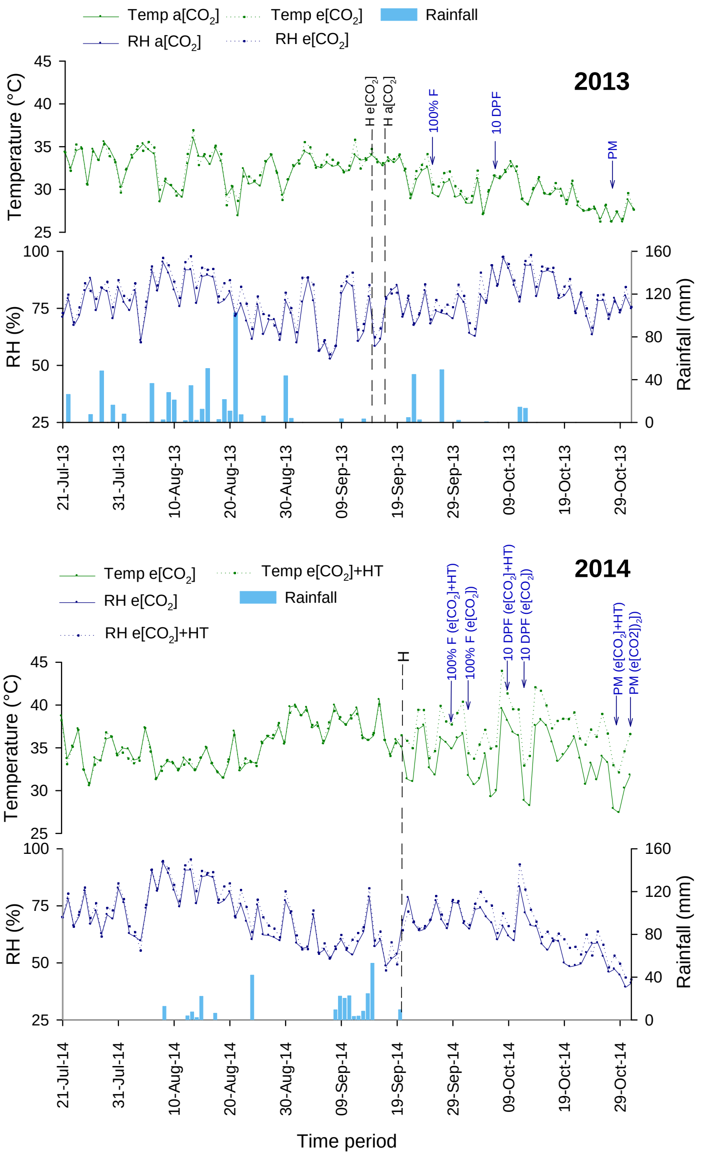

Supplementary Figure S1. Temperature, relative humidity and rain fall data recorded for the rice experiments during 2013 and 2014. Elevated CO_2 exposure during both year experiments was initiated at panicle initiation and continued until physiological maturity. High temperature exposure during 2014 was initiated from heading until physiological maturity. Phenology of both the cultivars was similar except 1-2 days delay observed in heading of Pusa 1121 across the experiments and treatments. However, e[CO_2] and e[CO_2]+HT treatments resulted in earlier heading, flowering and physiological maturity across the cultivars or experiments. Dotted vertical line shows heading (H) date for both the cultivars as observed under a[CO_2] and e[CO_2], respectively. Vertical descending arrows denote 100% flowering (100% F) and 10 days post flowering (10 DPF) when gas exchange traits and biochemical sampling was done across the cultivars and experiments. [Tavg.= average day time temperature (0700 to 1700 hrs); RH= average day time relative humidity; H= heading; 100%F= 100% panicle at flowering; 10DPF= 10 days post 100% flowering]

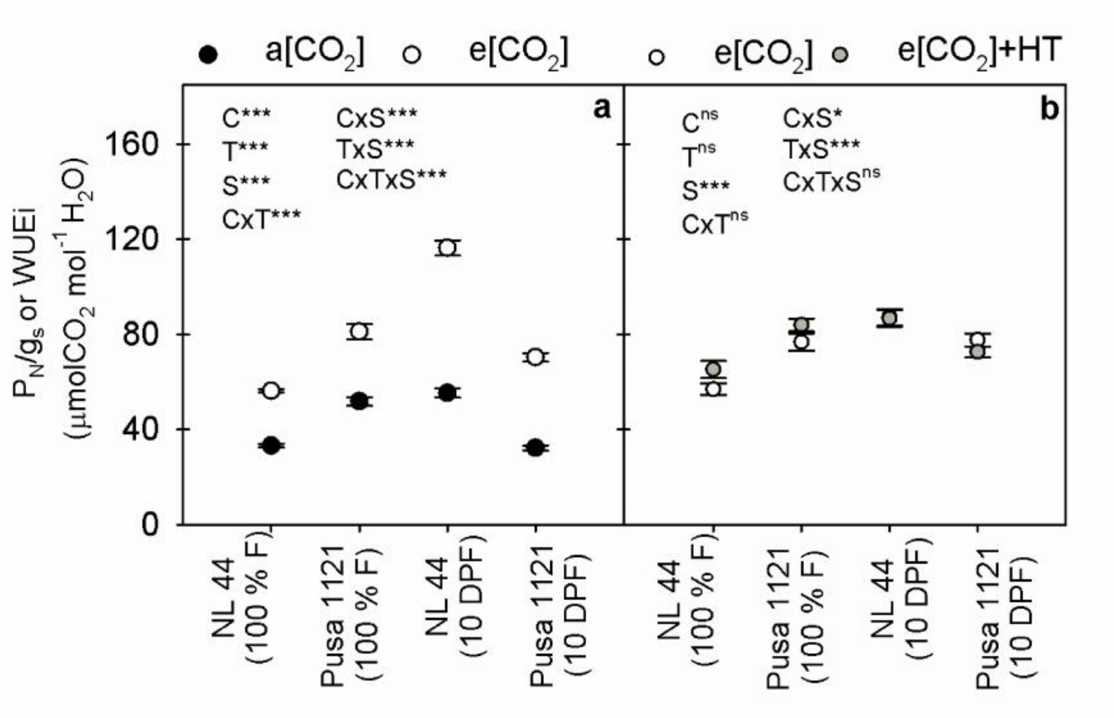

Supplementary Figure S2. Changes in intrinsic water use efficiency (WUEi; P_N/g_s) in flag leaf of rice cultivars NL-44 and Pusa-1121 at 100% flowering (100 % F) and at 10 days post 100% flowering (10 DPF) under ambient and elevated [CO_2] during 2013 (a) and elevated [CO_2] and elevated [CO_2] + HT environment during 2014 (b). Each circle represents mean of five replicates. Bars indicate ± SE. Significance level: **P<0.05*, ****P<0.001*, ns = non-significant. [C=cultivar; T= treatment; S= growth stage]

Supplementary Table S1. *ANOVA* table for leaf gas exchange parameters of rice cultivars NL-44 and Pusa 1121 measured at 100 % flowering (100% F) and 10 days post 100% flowering (10 DPF) under ambient and elevated [CO_2] during 2013 and elevated [CO_2] and elevated [CO_2] + HT environment during 2014. Values presented are least significant difference (LSD) for each trait.

**Source of variation**	**P_N**	**g_s**	**E**
2013			
Cultivar	0.505***	0.010***	0.109***
Treatment	0.505***	0.010***	0.109***
Stage	0.505***	0.010***	0.109***
Cultivar x Treatment	0.714^{ns}	0.015***	0.154***
Cultivar x Stage	0.714^{ns}	0.015***	0.154***
Treatment x Stage	0.714***	0.015*	0.154***
Cultivar x Treatment x Stage	1.010***	0.021^{ns}	0.218***
2014			
Cultivar	0.651***	0.016***	0.229***
Treatment	0.651***	0.016***	0.229***
Stage	0.651***	0.016***	0.229***
Cultivar x Treatment	0.92*	0.022^{ns}	0.325**
Cultivar x Stage	0.92^{ns}	0.022**	0.325^{ns}
Treatment x Stage	0.92***	0.022***	0.325***
Cultivar x Treatment x Stage	1.301^{ns}	0.031^{ns}	0.459^{ns}

P_N= photosynthetic rate, **g_s**= stomatal conductance, **E**= transpiration rate. Significance level: *$P<0.05$, **$P<0.01$, ***$P<0.001$, ns=nonsignificant

Supplementary Table S2. *ANOVA* table for total soluble sugars and starch content from leaf, stem and panicle of rice cultivars NL-44 and Pusa 1121 measured at 100 % flowering (100% F) and 10 days post 100% flowering (10 DPF) under ambient and elevated [CO_2] during 2013 and elevated [CO_2] and elevated [CO_2]+HT environment during 2014. Values presented are least significant difference (LSD) for each trait.

Source of variation	Total soluble sugars	Starch content
2013		
Cultivars (C)	0.39^{ns}	0.97^{ns}
Treatment (T)	0.39***	0.97***
Tissue type (TT)	0.48***	1.19***
Stage (S)	0.39***	0.97***
C x T	0.55*	1.37^{ns}
C x TT	0.68^{ns}	1.68*
T x TT	0.68***	1.68***
C x S	0.55^{ns}	1.37^{ns}
Tx S	0.55***	1.37*
TT x S	0.68***	1.68***
C x T x TT	0.96*	2.38^{ns}
C x T x S	0.78^{ns}	1.94^{ns}
C x TT x S	0.96**	2.38^{ns}
T x TT x S	0.96***	2.38***
C x T x TT x S	1.36^{ns}	3.36^{ns}
2014		
Cultivars (C)	0.35**	0.82***
Treatment (T)	0.35***	0.82***
Tissue type (TT)	0.42***	1.00***
Stage (S)	0.35***	0.82***
C x T	0.49***	1.16***
C x TT	0.60^{ns}	1.41^{ns}
T x TT	0.60***	1.41***
C x S	0.49***	1.16^{ns}
T x S	0.49**	1.16***
TT x S	0.60***	1.41***
C x T x TT	0.85**	2.00*
C x T x S	0.69^{ns}	1.63^{ns}
C x TT x S	0.85*	2.00^{ns}
T x TT x S	0.85^{ns}	2.00***
C x T x TT x S	1.20^{ns}	2.83**

Significance level: *$P<0.05$*, **$P<0.01$*, ***$P<0.001$*, ns=non significant

Supplementary Table S3. *ANOVA* table for sink enzymes activity in the spikelets of rice cultivars NL-44 and Pusa 1121 measured at 100 % flowering (100% F) and 10 days post 100% flowering (10 DPF) under ambient and elevated [CO_2] during 2013 and elevated [CO_2] and elevated [CO_2] + HT environment during 2014. Values presented are least significant difference (LSD) for each trait.

Interaction	CWI	VI	CI	SuSy	ADPGPPase	SSS
2013						
Cultivars	2.64^{ns}	0.51**	2.83***	5.74^{ns}	4.64**	10.56***
Treatment	2.64***	0.51***	2.83***	5.74***	4.64***	10.56***
Cultivars x Treatment	3.74*	0.72^{ns}	4.00*	8.12^{ns}	6.56**	14.93*
2014						
Cultivars	5.56^{ns}	1.09^{ns}	2.36***	5.03^{ns}	5.49^{ns}	14.42***
Treatment	5.56*	1.09^{ns}	2.36***	5.03***	5.49***	14.42**
Cultivars x Treatment	7.87**	1.54*	3.34***	7.12*	7.76**	20.39^{ns}

CWI = Cell Wall Invertase, **VI** = Vacuolar Invertase, **CI** = Cytosolic Invertase, **SuSy** = Sucrose Synthase, **ADPGPPase** = ADP-Glucose Pyrophosphorylase, **SSS** = Soluble Starch Synthase.
Significance level: *$P<0.05$, **$P<0.01$, ***$P<0.001$, ns=non significant
